# Testing Chemicals for Effects on Breast Development, Lactation, and Cancer

**DOI:** 10.1289/ehp.1104077

**Published:** 2011-08-01

**Authors:** Julia Green Brody, Ruthann A. Rudel, Mhel Kavanaugh-Lynch

**Affiliations:** Silent Spring Institute, Newton, Massachusetts, E-mail: brody@silentspring.org; California Breast Cancer Research Program, University of California, Office of the President, Oakland, California

Factors across the life cycle, beginning before birth, influence breast cancer risk. We know this from epidemiologic studies of characteristics self-reported by older women or gleaned from medical records. For example, larger babies have higher breast cancer risk decades later, and preeclampsia is associated with lower risk in daughters (Ruder et al. 2008; Xue and Michels 2007). Earlier puberty increases risk, and women with fewer or no pregnancies have higher risk later on (Bernstein 2002). These factors span sensitive periods of breast development, prenatally and during puberty and pregnancy (Rudel et al. 2011).

If environmental chemicals have comparable effects during sensitive developmental periods, identifying these risks could lead to prevention. However, epidemiologic data to assess the effects of chemicals in early life are rarely attainable. Exceptional events or decades-long cohort studies can provide some information. For example, studies of atom bomb survivors revealed breast cancer risk was highest from radiation exposure to young girls (Land 1995). A unique study using blood collected in the 1960s and stored for more than 40 years found higher breast cancer risk in women who were younger than 14 years of age when DDT was first put into use, taking advantage of a natural “experiment” (Cohn et al. 2007). But we cannot rely on observational chances like these to evaluate breast cancer risks for the many chemicals in use today.

Thus, improving test methods in animals and cells is essential to identify chemicals that may interfere with breast development and contribute to cancer, so we can use this knowledge for primary prevention. These methods will extend recent changes in toxicity testing, which were designed to respond to research on endocrine-disrupting compounds by evaluating how exposures *in utero* and during other windows of development set the stage for chronic diseases later in life. New procedures include dosing *in utero* and throughout development, adding morphological and functional assessments of reproductive organs, and including longer-term follow-up, none of which are included in traditional toxicity testing. For example, new protocols recently demonstrated that *in utero* antiandrogen exposure in rodents leads to altered male reproductive development, as well as reduced fertility and possibly increased cancer later on. These observations are thought to parallel human “testicular dysgenesis syndrome,” which includes decreased fertility and increased incidence of cryptorchidism, hypospadias, and testicular cancer (Luccio-Camelo and Prins 2011).

Mammary gland development has usually not been assessed in the new protocols, though. Yet, mounting evidence supports the importance of testing for breast effects for several reasons. The breast develops over a long period, with vulnerability beginning *in utero* and extending through the first pregnancy. For some chemicals tested to date, mammary gland development in males and females is altered at lower doses than the levels that cause changes in other tissues. The effects that have been observed after disrupted mammary gland development include impaired lactation and increased susceptibility to cancer, so they are of potentially great public health significance.

In this issue, three articles address these points:

Makris (2011) reviews standard chemical testing protocols to identify gaps in mammary gland assessment. Key gaps include lack of early life exposure in tests for carcinogenic effects; lack of assessment of lactation function other than nonspecific measures; and inadequate examination of mammary gland morphology and pathology.

Rudel et al. (2011) present a comprehensive review of hormone and chemical effects on mammary gland development, lactation, and cancer. Emerging from a meeting of 60 international experts, this article reports the majority opinion of this group that normal mammary gland development and carcinogenesis are similar in rodents and humans; that chemical and hormone exposure *in utero* or early in life leads to altered mammary gland development; and that these changes may be risk factors for impaired lactation and cancer. Effects on mammary gland development are not limited to estrogenic endocrine disruptors but are induced by diverse chemicals, including perfluorinated compounds and the herbicide atrazine, in addition to the soy phytoestrogen genistein and synthetic estrogens such as bisphenol A.

White et al. (2011) demonstrate persistent effects of perfluorooctanoic acid (PFOA) in drinking water on mouse mammary gland development at exposures lower than in some contaminated drinking water supplies.

Research needs identified in the articles include a call for dosing during development; improved assessment of mammary gland development, structure, and function; and increased assessment of the male mammary gland. These recommendations have been implemented by the National Center for Toxicological Research in several studies done in conjunction with the U.S. National Toxicology Program (NTP) (Delclos et al. 2009; Latendresse et al. 2009), but they still need to be integrated into standard protocols at the NTP, the U.S. Environmental Protection Agency (EPA), and the Organisation for Economic Co-operation and Development (OECD). Research is also needed to determine the relationship of mammary gland changes to effects on lactation and cancer susceptibility. The $5 million California Breast Cancer Research Program initiative “Making Chemicals Testing Relevant to Breast Cancer” is a model for stimulating research in this field, and the National Institute of Enironmental Health Sciences (NIEHS) Breast Cancer and the Environment Research Program is contributing knowledge about puberty as a critical window of development and susceptibility.

Although traditional cancer bioassays have identified many common pollutants that increase mammary gland tumors, including common air pollutants, drinking water disinfection by-products, and chlorinated solvents (Rudel et al. 2007), research reported in these three articles suggests that traditional tests that neglect developmental effects may be missing many more. Given the magnitude of potential public health impacts on breast-feeding and breast cancer, it is critical to strengthen testing methods and give more weight to them in policy decisions. Good decisions about pollution limits, pesticide approvals, and chemicals in consumer products and food rely on a full and accurate understanding of risks associated with exposure.
